# Using antimicrobial adjuvant therapy in cancer treatment: a review

**DOI:** 10.1186/1750-9378-7-33

**Published:** 2012-11-20

**Authors:** Kenneth Alibek, Aliya Bekmurzayeva, Assel Mussabekova, Bolat Sultankulov

**Affiliations:** 1 Nazarbayev University, 53 Kabanbay Batyr Avenue, Astana 010000, Kazakhstan; 2 Republican Scientific Center for Emergency Care, 3 Kerey and Zhanibek Khanov Street, Astana 010000, Kazakhstan; 3 “Nazarbayev University Research and Innovation System” private institution, Nazarbayev University, 53 Kabanbay Batyr Avenue, Astana 010000, Kazakhstan

**Keywords:** Cancer, Antibiotic, Antiviral, Chemotherapy, Bacteria, Virus, Apoptosis, Neutropenia

## Abstract

Recent clinical and pre-clinical data demonstrate that adjuvant antimicrobial therapy is beneficial in cancer treatment. There could be several reasons for this effect, which include treating cancer associated bacteria and viruses, prophylaxis of post-chemotherapy infections due to immunosuppression, and antiproliferative effect of certain antimicrobials. Targeting cancer associated viruses and bacteria with antimicrobial agents is currently used for gastric, cervical, hematopoietic, liver and brain cancer. However this treatment is effective only in combination with conventional therapies. Antimicrobials can also have a direct antiproliferative and cytotoxic effect, and can cause apoptosis. Moreover, some antimicrobials are known to be helpful in overcoming side effects of drugs commonly used in cancer treatment. Chemotherapy related bacteremia and neutropenia can be overcome by the appropriately timed use of antimicrobials. This review summarizes the data on the effects of antivirals and antibiotics on cancer treatment and describes their mechanisms.

## Introduction

Major conventional cancer therapies include surgery, chemotherapy, radiation therapy and symptom care. Since 2010 cancer vaccines have been approved for use against hepatitis B virus-associated liver cancer and human papilloma virus-associated cancers. These therapies have contributed to increased survival rates. Additional targeted therapies have been approved by the Food and Drug Administration (FDA) [1]. However, despite recent advances in treatment methodologies, there is still significant mortality rate associated with most cancers. There is compelling data to support the clinical use of antiviral and antibacterial agents in combination with existing cancer therapies. Support for the use of adjuvant antimicrobial therapy includes the existence of cancer-associated viruses and bacteria, antimicrobial prophylaxis following chemotherapy-associated immunosuppression and anti-proliferative activity of certain antimicrobials. This review summarizes clinical data on the use of antivirals and antibiotics in cancer therapy and discusses possible mechanisms for their contribution to cancer treatment.

### Using antimicrobials to treat cancer-associated infections

More than 100 years have passed since it was first proposed that infections may cause cancer [2], however the role of viruses and bacteria in cancer is highly underrated among the cancer research community. Currently identified oncogenic viruses include Hepatitis B virus (HBV) and human papilloma virus (HPV), against which global vaccination programs have been introduced. Other oncogenic viruses include hepatitis C virus (HCV), human T-lymphotropic virus (HTLV) and members of *Herpesviridae* family, including Epstein-Barr virus (EBV), Kaposi-sarcoma herpesvirus (KSHV) and cytomegalovirus (CMV) (reviewed in [2,3]). Epidemiological evidence also shows strong association between certain bacteria (*Helicobacter pylori, Salmonella typhi*) and cancer (reviewed in [4]). The roles and/or mechanisms of many other pathogens (polyomavirus, JC and BK polyomaviruses, *Chlamydia* spp., *Mycoplasma* spp., *Propionibacterium* acnes, *Streptococcus anginosus* and other) are still unknown. Growing evidence that infectious agents are factors in tumorogenesis supports the use of antimicrobial therapy in certain pathogen-associated cancers. Herein, we give examples of clinical studies which demonstrate the use and efficacy of antimicrobial adjuvant therapy.

#### Hepatocellular carcinomas

Up to 80% of hepatocellular carcinomas (HCC) are associated with chronic HBV and HCV infections. This suggests that almost 500,000 deaths could be prevented annually by antiviral therapies. Clinical data show that vaccination against HBV and antiviral therapy against HCV can slow down the progression of liver disease (reviewed in [5]). However, antiviral efficacy is significantly better in patients during early stages of infection. It was shown [6] that oral antiviral therapy reduces the risk of hepatocellular carcinoma only in patients without cirrhosis, but could not protect patients with chronic infection.

#### Brain tumors

One of the members of *Herpesviridae* family, CMV is considered an important therapeutic target for treating brain tumors [7]. Human CMV (HCMV) has been shown to induce the expression of COX-2, growth factor for tumor cells. Many tumors express COX-2, moreover, direct or indirect inhibition have been shown useful in treating cancers. The HCMV protein US28 induces COX-2 and Wnt target gene expression, which leads to accumulation of β-catenin thereby increasing proliferation. HCMV is commonly associated with brain tumor as it was present in human medulloblastoma cell lines, and HCMV proteins were expressed in human brain tumors [7]. A combination of ganciclovir (an antiviral drug) and celecoxib (COX-2 inhibitor) reduced the proliferative capacity of HCMV positive cell lines but had no effect on HCMV negative medulloblastoma cells. This drug combination significantly reduced tumor growth *in vitro*. Use of the same combination of drugs in murine model with HCMV-positive cancer cells led to a 72% tumor reduction. These studies show that the effect of the antiviral drug was HCMV specific.

Another promising antiviral drug for HCMV-associated glioblastoma treatment is acyclovir, which inhibits HCMV thymidine kinase [8]. It was reported that T-cell therapy for patients with CMV-associated glioblastoma was beneficial. They expanded CMV-specific T-cell population, specific for viral pp65+ and IE1+ targets, inhibited CMV-infected tumor cells [9].

#### Human herpes virus 8 associated cancers

KSHV (human herpes virus 8 [HHV8]) was shown to be significantly associated with Kaposi’s sarcoma (KS), primary effusion lymphoma (PEL), and Castleman’s disease. In preclinical studies, HHV-8 was sensitive to ganciclovir, cidofovir and foscarnet but resistant to cyclovir in preclinical studies. While several retrospective studies show prophylactic usefulness of ganciclovir or foscarnet against KS in patients with AIDS, other studies did not demonstrate the same results. The role of antivirals in therapies for Kaposi’s sarcoma is inconclusive due to the lack of clinical follow-up and small number of patients in these studies [10-12]. Tumor cell line models for PEL can be targeted for apoptosis by inducing the activation of latent HHV-8 infection. In this case, valproate was used to trigger lytic replication of the virus, whereas antiviral drugs were used to effectively control the activated virus. Further studies would prove useful to investigate whether the combination of valproate and antiviral agents will be beneficial in a clinical setting. Another severe lympho-proliferative complication in HIV positive patients is Castleman’s disease. Ganciclovir improved outcomes in three HHV-8 and HIV-positive patients with Castleman’s disease and decreased HHV-8 DNA levels [13].

#### Nasopharyngeal carcinoma

Nasopharengeal carcinoma (NPC) is associated with several viruses, such as HPV and EBV. The latter was ranked as a group 1 carcinogenic agent by the International Agency for Research Cancer [14]. It was found that [15] found that cidofovir injected into tumor sites inhibited tumor growth and EBV. In addition, as reviewed in [16], cidofovir decreased tumor size and induced apoptosis in HPV-infected nasopharyngeal carcinoma xenografts of athymic mice. EBV DNA was detectable in more than 90% of plasma samples from patients with NPC, compared to 7% in healthy control patients. In addition, it was shown that EBV DNA levels can be used to predict clinical outcomes of the treatment in NPC patients. Although NPC is associated with infection additional clinical data should be acquired in order to determine if NPC can be prevented and/or treated using other antiviral therapies.

#### Hematopoietic cancers

Viral infections with HCV, EBV and human T-cell lymphotropic virus type I -HTLV1 are associated with hematopoietic cancers. There are several clinical studies showing that zidovudine treatment, in combination with interferon α (IFN-α), targets EBV in lymphoma cells [16,17] and HTLV1 in adult T-cell leukemia-lymphoma [18], leading to tumor inhibition. Interestingly, zidovudine alone has poor antitumor activity because it is a poor substrate for mammalian DNA polymerases. However, when used in combination with inhibitors of *de novo* synthesis of thymidylate (e.g. MTX, IFN) it can have cytotoxic effect against tumors. The direct cytotoxic effect of zidovudine and IFN were not found in *in vitro* studies with HTLV1 positive cells. These results, together with studies showing zidovudine’s inhibition of HTLV1 replication, and transformation of healthy lymphocytes by HTLV1 *in vitro,* suggest a direct anti-viral activity of this drug during treatment of adult T-cell leukemia. Moreover, there is some evidence of direct antiviral effect of IFN alone, against HTLV1 [19]. Treating EBV infection in central nervous system lymphoma was successful in some cases, where a combination of rituximab (an anti-CD20 monoclonal antibody) and antiviral drugs were used [18-20]. Full remission of acute lymphoblastic leukemia after HCV-targeted treatment with the combination of peginterferon α 2a and ribavirin was shown in the clinical case was reported [21].

#### Cancers associated with bacteria

As reviewed by [4], infections caused by several bacteria (e.g. *Bartonella spp., Lawsonia intracellularis* and *Citrobacterrodentium*) can induce cellular proliferation that can be reversed by antibiotic treatment. Antibiotic therapy is common practice in mucosa associated lymphoid tissue (MALT) lymphoma, gastric cancers and cervical cancers. Population study on cervical cancer by Tamim et. al [22] has shown the association of cancer development with a *Neisseria gonorrhoeae* and *Chlamydia trachomatis* infections and exposure to antibiotics up to 15 years in the past was associated with a decreased risk of cervical cancer development.

*Helicobacter pylori* is a well-recognized agent involved in gastric MALT lymphomas and gastric carcinomas [23,24] and is increasingly associated with eye cancer, breast cancer, and lung cancer [22]. The FDA and World Health Organization (WHO) recommend the use of a combined therapy to treat *Helicobacter pylori* infection: amoxicillin and clarithromycin together with proton pump blockers such as omeprazole/lanzoprazole and muco-protectants (e.g. Sucralfate) [25]. Elimination of bacterial infection leads to partial or complete remission of MALT lymphoma in 60−80% of patients [23]. A review of 24 studies showed complete remission of MALT lymphoma in 35−100% of patients [26]. Thus, antibiotic therapy is recommended as an affordable and safe first line treatment, especially in low-grade MALT lymphomas, while oncologic therapies are recommended for patients not responding to antibiotic therapy [26]. Similarly, *Chlamydia psitacii* eradication has been recommended instead of more aggressive treatment regimens for treatment of MALT-type eye cancers [22].

Overall, it can be concluded that in the cancers which are strongly associated with infectious pathogens, antimicrobial therapy shows promising results. However, almost always it is helpful only in combination with existing chemotherapies and immunomodulatory substances.

### Antimicrobials used as anti-proliferative and cytotoxic agents

**Figure 1:**
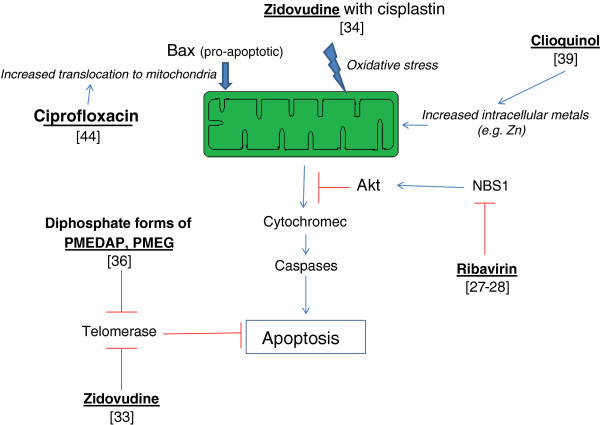
Schematic representation of the effect of antivirals and antibiotics on apoptosis.

In addition to the proposed use of antiviral and antibiotic agents in treatment of cancers associated with infections, these agents were reported to have direct cytostatic and cytotoxic activities in cancer (both *in vitro* and *in vivo)*. Antiproliferative and cytotoxic effects of selected antivirals and antibiotics are shown in Table 1. Examples of the mechanisms by which some antivirals and antibiotics can cause apoptosis are shown in Figure 1.

**Table 1:** Antiproliferative and cytotoxic effect of selected antivirals and antibiotics

**Anti-microbial**	**Target and its function**	**Mechanism**	**Shown in**	**References**
**Ribavirin**	Eukaryotic translation initiation factor: **eIF4E**; Enhanced mRNA transport and translation of cyclin D1, survivin, c-myc, VEGF and etc.	Ribavirin binds to eIF4E with high affinity and competes with its binding to mRNA; selectively disrupts eIF4E subcellular organization and therefore transport and translation of mRNAs which are post-transcriptionally regulated by eIF4E leading to decreased levels of oncogenes such as cyclin D1.	Patients with acute myeloid leukemias; Breast cancer cell lines	[27-29]
**Acyclovir**	indoleamine 2,3-dioxygenase (**IDO**); enhances activity of Treg while Treg inhibits immunity in glioblastomas	Acyclovir could decrease Treg function in glioblastoma and could potentially be used as an adjunct in therapy	Proposed	[8]
**17-AAG, 17-DMAG (geldanamycin analogs)**	**HSP-90** promotes survival of tumor cell, induces their growth and metastasis even when there are no growth factors via continued protein translation and cellular proliferation; Its client proteins include: KIT, AKT, CDK4, telomerase, Bcl-2, MMP2 and others.	Inhibition of HSP90 leads to decreased level and activity of its client protein protooncogene KIT and downstream signaling molecules AKT and STAT3	HMC-1 cells derived from a patient with mast cell leukemia	[30,31]
		Hsp90 inhibition by 17-AAG leads to decreased proliferation and viability and increased radiosensitivity of cancer cells	Oesophageal squamous cell carcinoma cell lines	[32]
**Clioquinol**	Acts as metal **ionophore**	Probably increases metal concentration in mitochondria and induces release of cytochrome c leading to apoptosis	DHL-4, A2780, SiHa and other cells and mouse xenograft model	[33]
**PMEG, PMEDAP**	**VEGF, EGF, FGF, PDGF and their receptors** are important in angiogenesis	Diphosphate derivatives of PMEG and PMEDAP inhibit DNA polymerase and activity of human telomerase in vitro. DNA damage could affect signalling pathways associated with angiogenesis.	SD-lymphoma bearing rats	[34]

#### Antivirals used as anti-proliferative and cytotoxic agents

Many antiviral agents were reported to have anti-proliferative and proapoptotic activity in various cancers through inhibition of transcriptional factors, inhibition or activation of human DNA polymerase, suppression of telomerase activity, increases in radiosensitivity and downregulation of angiogenic genes. It was found that certain antiviral agents can target translation initiation factors. eIF4E, a eukaryotic translation initiation factor, which is elevated in 30% of cancers, binds 7-methyl guanosine cap on mRNA enhancing nuclear RNA transport and translation. Treatment with the broad spectrum antiviral agent ribavirin was beneficial in poor prognosis acute myeloid leukemia [27]. Ribavirin relocalizes eIf4E to the cytoplasm. By competing with binding of eIF4E with mRNA and disrupting structure of eIF4E, ribavirin reduces oncogene (cyclin D1 and NBS1) levels [27,28]. eIF4E is also known to regulate the expression of such important molecules in cancer development as vascular endothelial growth factor (VEGF), survivin, c-myc [29]. Ribavirin also inhibited eIF4E-associated transformation of cells in vitro and eIF4E-dependent squamous cell carcinoma of human in in vivo mouse model [28]. In a similar study, ribavirin inhibited cell proliferation, reduced expression of eIF4E in breast cancer cell line[29]. Ribavirin also modulates immunity and targets inosine monophosphate dehydrogenase, enzyme in guanosine biosynthesis pathway which may be an alternative explanation of its antiproliferative activity [35].

It is known that inhibition of T-regulatory cells (Treg) results in improved cancer immunotherapy [36]. Glioblastomas are characterized by a mild state of systemic immunosuppression. Together with its potential use in glioblastoma treatment due to its ability to inhibit HCMV thymidine kinase another mechanism by which acyclovir could be used in cancer therapy is by Treg inhibition in glioblastomas. It has been shown that acyclovir has an inhibitory activity on indoleamine 2,3-dioxygenase, and thereby could inhibit Treg function. Given the short survival time of patients with glioblastoma together with relative safety of acyclovir, using this antiviral as an adjunct therapy could have a potential in glioblastoma treatment [8].

Cancer cells are characterized by increased telomerase activity and telomerase inhibition leads to apoptosis [37]. Zidovudine, an antiviral drug, was shown to suppress telomerase activity and increase the radiosensitivity in glioma cells [38]. In another study, zidovudine as an inducer mitochondrial dysfunction was shown to enhance cytotoxic activity of cisplastin via thiol metabolism and oxidative stress in cells of head and neck cancer. Zidovudine’s effect against cancer cells was due to causing increased oxidative stress [39]. The viability of human hepatoma cells was significantly decreased after four week treatment with zidovudine (AZT) and one week recovery. This was due to a delay of cell cycle progression, induction of apoptosis and decreased telomerase activity [40].

Diphosphate forms of PMEG **(**9-[2-(phosphonomethoxy)ethyl]-guanine) and PMEDAP (9-[2-(phosphonomethoxy)ethyl]-2,6-diaminopurine) have ability to inhibit human telomerase activity in vitro. Another prospective use of antivirals lies in their ability to inhibit angiogenic genes. For instance, in rats with SD-lymphoma PMEG and PMEDAP were able to modulate expression of angiogenic genes. PMEG was more potent in downregulation of expression of such genes as epidermal growth factor (EGF), fibroblast growth factor (FGF), platelet-derived growth factor (PDGF), vascular endothelial growth factor (VEGF) and their receptors while PMEDAP affected mainly expression of VEGF and its receptor [34].

#### Antibiotics used as anti-proliferative and cytotoxic agents

Anthracycline antibiotics such as doxorubicin and daunomycin are widely used in cancer treatment in humans [41]. Although the exact "antitumor activity of anthracycline" is controversial, suggested mechanisms include: intercalation into DNA, production of free radicals, DNA binding and alkylation, DNA cross-linking, inhibition of topoisomerase II. However the amount of doxorubicin used during some of the studies was supraclinical complicating the proposed mechanisms of antitumor activity. Anthracyclines are also known to induce cardiomyopathy [42]. Other classes of antibiotics are under investigation for their potential application in cancer treatment and mode of antiproliferative action is various in different classes of antibiotics.

After discovering that some metal chelators such as desferrioxamin can inhibit tumor growth, other antibiotics chelators were investigated for their potential antitumor abilities. Clioquinol as a chelator of copper and zinc was hypothesized to inhibit superoxide dismutase-1, a potential tumor target, which uses these metals as cofactors. Clioquinol treatment led to apoptosis via caspase activation in human tumor cell lines. However, cytotoxicity of this agent was not due to its chelating abilities because addition of copper and zinc enhanced its antitumor effect but this antibiotic had an ability to transport metals inside the cell and act as a transition metal ionophore instead. Increased concentrations of metals in mitochondria, for example, can lead to Cytochrome c release leading to apoptosis [33].

A number of thiazole antibiotics were revealed to have antiproliferative activity by inhibiting transcription factors. It was shown in *in vitro* experiments that thiazole antibiotics Siomycin A and thiostrepton inhibit transcriptional activity of Forkhead box M1 or FoxM1. FoxM1 is an oncogenic transcription factor and considered to be a promising target in tumor treatment since it is overexpressed in many types of human tumors such as hepatocellular carcinomas, pancreatic carcinomas, breast cancers, non-small cell lung carcinomas. Particularly these antibiotics had antiproliferative activity and can induce apoptosis [43]. This family of antibiotics is known to bind to the large ribosomal subunit and inhibit protein synthesis in prokaryotes. Furthermore thiazole antibiotics had anti-proliferative activity in vivo in xenograft model of human breast cancer [44].

A number of fluoroquinolones were shown to have antitumor activity as well [45]. In bacterial cells they inhibit topoisomerase type II/DNA gyrase [46] and until recently their effect on mammalian cells was unknown. One of fluoroquinolones, ciprofloxacin, inhibited the growth of bladder transitional cell carcinoma, colon cancer and prostate cancer cells [45]. It induced apoptosis and inhibited proliferation of human colorectal carcinoma cells presumably by blocking mitochondrial DNA synthesis. Ciprofloxacin had no such an effect on hepatoma cells [46]. Another member of this family of antibiotics moxifloxacin had an ability to inhibit activation of NFkB, mitogen-activated protein kinase and interleukin-8 synthesis. Moxifloxacin enhances etopisode’s (VP-16) antitumor effect in tumor cells (THP-1 and Jurkat) via inhibition of topoisomerase II. Moreover, moxifloxacin overcomes one of the drawbacks of using etopisode in cancer treatment by it inhibiting the release of proinflammatory cytokines [45]. Ciprofloxacin induced apoptosis and reduced proliferation of prostate cancer cells whereas non-tumorigenic prostate epithelial cells were not affected. The mechanism of ciprofloxacin activity is associated Bcl-2- associated X protein (Bax) insertion into mitochondrial membrane, increased ratio of Bax-to-Bcl-2 and cell cycle arrest at S-G2/M phase [47].

Some members of benzoquinanone ansamycin such as geldanamycin and its derivatives (17-AAG, 17-DMAG) cause degradation of tumorigenic proteins by binding to heat shock protein 90 (Hsp90) [30]. Hsp90 promotes survival of tumor cell, induces their growth and metastatsis even when there are no growth factors via continued protein translation and cellular proliferation. Client proteins of Hsp90 important in cancer which include various transcription factors, kinases and etc are reviewed elsewhere [31]. 17-AAG can result in destabilization of Hsp-dependent kinases that are important in cancer development by inhibiting Hsp90 [30]. The geldanamycin derivative,17-AAG had antiproliferative and cytotoxic activity human oesophageal cancer cell lines and increased their radiosensitization [32].

### Antimicrobial prophylaxis prior and after cancer therapy

#### Chemotherapy-induced neutropenia and bacteremia

Patients suffering from malignant tumors often develop chemotherapy-induced neutropenia leading to immunosuppression and bacterial, viral and fungal infections. Ten to fifty percent of patients with solid tumors and 80% of those with hematologic malignancies will develop fever associated with neutropenia during more than one chemotherapy cycle [48]. Infections in patients suffering from hematological malignancies are a major problem. Malignancies by themselves, or by virtue of their therapeutic strategies (chemotherapy, radiation, hematopoietic stem cell transplant [HSCT]), put patients at risk for infections. Some infections are associated with specific predisposing factors in patients with hematological malignancies, such as neutropenia with insufficient phagocytosis, abnormal T-cells with cellular immune dysfunction, abnormal B-cells with humoral immune dysfunction and chemotherapy-related immune suppression [49]. Abnormal T-cells are associated with higher risks for *Listeria*, *Legionella*, *Salmonella*, *Nocardia*, *Pneumocytis*, *Toxoplasma*, *Cryptosporidium*, *Mycobacteria*, *Candida*, *Cryptococcus*, *Histoplasma,* and HHV infections. Hematological patients with abnormal B cells with humoral immune dysfunction are at increased risk of sinusitis, pneumonia, meningitis and sepsis [50].

Bacteremia was documented in about 10%-25% of febrile neutropenia cases [51]. Common infection sites include tissue-based infection of the intestinal tract, lung, and skin. Therefore, initiation of antibiotic therapy after chemotherapy is vital for preventing mortality due to infections [52]. During the initial development and practical implementation of chemotherapy in 1960s and 70s, Gram-negative pathogens predominated following chemotherapy treatments. Currently Gram-positive infections are becoming more common in cancer patients. The latter include coagulase-negative staphylococci, such as *Staphylococcus aureus*, including methicillin-resistant strains, *Enterococcus* species, including vancomycin-resistant strains S.viridans streptococci, *Streptococcus pneumoniae*, *Streptococcus pyogenes*. Common Gram-negative bacteria include *Escherichia coli*, *Klebsiella* species, *Enterobacter*species, *Pseudomonas aeruginosa*, *Citrobacter*species, *Acinetobacter*species, *Stenotrophomonas maltophilia*[53,54]*.* The increasing number of Gram-positive infections is probably due to the increased use of indwelling plastic venous catheters which allow the entry of Gram-positive bacteria from the skin.

Drug-resistant Gram-negative infections are predominating in neutropenic patients [55-57]. Broad spectrum beta-lactamase genes are often expressed by *Klebsiella* species and *E.coli* strains, and result in β-lactam antibiotic resistance [58,59]. Resistance among Gram-positive pathogens especially methicillin resistant *Staphylococcus aureus* and vancomycin resistant enterococci have become more common, accounting for about 40% [60,61].

Colony stimulating factors (CSFs) are used as a prophylactic agent in cancer patients suffering from chemotherapy-induced neutropenia. It has been demonstrated [62] that administration of antibiotics such as levofloxacin, ciprofloxacin and norfloxacin with addition to granulocyte CSF decreased the number of identifiable infections in lung cancer patients. Preclinical data [63] has shown that the administration of antibiotics such as ciprofloxacin, trimethoprim-sulfamethaxazole, cafazolin and nitrofurantoin, after transurethral resection of bladder tumors could prevent seeding of cancer cells, as a result decrease the rate of recurrence in a dose dependent manner. Ciprofloxacin exhibit time- and dose dependent cytotoxicity against transitional carcinoma and significantly enhance cytotoxic effect of doxorubicin [64].

#### Fungal infections prophylaxis

Patients with hematologic malignancies and hematopoietic stem cell transplantation (HSCT) recipients represent a population at high risk for invasive fungal disease. Late fungal infection diagnoses are associated with severe morbidity and high mortality of hematological patients. In 2005, as a result of collaborations between several groups, including the European Group for Blood and Marrow Transplantation, the European Organization for Treatment and Research of Cancer, the European Leukemia Net and the Immuno-compromised Host Society, the European Conference on Infections in Leukemia (ECIL) was created. The main goal of ECIL is to elaborate upon guidelines or recommendations for the management of infections in hematology patients, including information on antifungal prophylaxis in high-risk hematology patients, empirical antifungal therapies, and treatment strategies for invasive *Candida* and *Aspergillus* infections.

Guidelines for primary antifungal prophylaxis are available [65,66]. In cases of allogeneic HSCT the ECIL-3 Working Group has proposed to provide phase-specific guidelines for HSCT recipients such that fluconazole is highly recommended in the initial phase, but only when combined with a mold-directed diagnostic approach. Posaconazole is the drug of choice at the onset of acute or chronic graft-versus-host disease (GVHD). However, the working group recommended therapeutic drug monitoring, especially in patients with intestinal GVHD. During GVHD, fluconazole becomes less relevant because of the high risk of mold disease. Voriconazole and itraconazole prophylaxis has also been shown to be as effective as fluconazole for *Candida* prophylaxis in patients undergoing allogeneic stem cell transplants [67-69]. According to published data [67,68,70] the ECIL 3 recommends use of voriconazole in both phases of HSCT (initial neutropenia and GVHD phases) with a provisional AI grading.

#### Viral infections prophylaxis

Agents against HSV such as acyclovir should be offered to HSV-seropositive HSCT recipients [71] and patients with acute leukemia [72] as a prophylaxis until recovery of the white blood count or resolution of mucositis. Prophylaxis can be extended for patients with frequent recurrent HSV or GVHD infections and could last for up to 1 year [73]. Other herpesvirus infections occur in the post-HSCT setting, including infections due to cytomegalovirus and human herpesvirus 6.All cancer patients should be immunized against influenza on a yearly basis, despite the illusive vaccine efficacy, inactivated vaccine are shown to yield adequate response in some patients treated for solid malignancies [74,75]. However, live attenuated influenza vaccines should be avoided in patients receiving chemotherapy cycles and during 6 months after the end of therapy. It is was shown that influenza vaccination responses may be best between chemotherapy cycles (7 days after the last treatment) or 2 weeks before chemotherapy starts [75-77]. HSCT recipients usually respond best to influenza vaccination if vaccinated at 6 months after transplantation. If an exposure to influenza occurs, 5 days of post-exposure treatment with anti-influenza antivirals (eg, oseltamivir or zanamivir) is recommended for the neutropenic patient regardless of vaccination status [78]. Patients with respiratory complaints, including cough and nasal congestion or a pulmonary infiltrate noted on chest radiograph during the peritransplant period, should be evaluated by examination of nasopharyngeal swab or washing specimens. The specimen can be tested by PCR, direct antigen assay, or culture for respiratory viruses (including influenza, parainfluenza, adenovirus, RSV, and human metapneumovirus) [79]. Neutropenic patients infected with respiratory viruses (including influenza, parainfluenza, adenovirus, RSV, and human metapneumovirus) may be a febrile and may lack “classic” systemic symptoms, such as myalgia [80]. Ribavirin has been used, however there is no antiviral agent proven to be effective against parainfluenza virus [81] and RSV pneumonia [82]. Monoclonal antibody (palivizumab) and RSV immunoglobulin are not preventing or attenuating RSV upper respiratory infection [83]. Cidofovir or ribavirin were used for clinically significant adenovirus disease [84], however there is no proven effective therapy against adenovirus.

Overall, chemotherapy-related immunosuppression resulting mainly in inhibition of neutrophils and subsequent infections is a significant problem in cancer treatment, where antimicrobial prophylaxis can be of a great clinical significance.

## Conclusion

Outcomes of cancer treatments depend on many factors, many of which are still unknown. Frequent treatment complications include viral, bacterial, and fungal infections, which have roles in the etiology, affect the outcome of the treatment, and may arise as a side effect of the therapy. With development of many classes of antimicrobials, microbial related carcinogenesis and therapy complications are the most preventable. However, there are not many studies that have investigated the value of adjuvant antimicrobial therapy in the course of cancer treatment.

Antimicrobial agents can benefit cancer patients by killing oncogenic-related microorganisms, by protecting from recurring immunosuppression-induced infection, and by their direct antiproliferative/cytotoxic effects. Different mechanisms are involved in antiproliferative effects of antimicrobials, such as targeting translation initiation factors, degrading tumorigenic proteins by binding to heat shock proteins involved in their folding, having antiangiogenic effects, inhibiting topoisomerases, destabilization of mitochondria and other effects causing apoptosis. Moreover, antimicrobials could potentially decrease some side effects of currently used therapies against cancer. These include inhibition of proinflammatory cytokine release and the induction of cytotoxic activities by conventional chemotherapy drugs. However, the current administration of antivirals and antibiotics in cancer therapy should only be in combination with anticancer drugs to increase the effectiveness of current therapies.

## Abbreviations

FDA: Food and Drug Administration; HBV: Hepatitis B virus; HCB: Hepatitis C virus; HPV: Human papilloma virus; EBV: Epstein-Barr virus; KSHV: Kaposi’s sarcoma herpes virus; CMV: Cytomegalovirus; HCMV: Human cytomegalovirus; COX-2: Cyclooxygenase 2; HHV-8: Human herpes virus 8; NPC: Nasopharyngeal carcinoma; HTLV1: Human T-cell lymphotropic virus type I; MTX: Methotrexate; MALT: Mucosa-associated lymphoid tissue; VEGF: Vascular endothelial growth factor; Treg: T-regulatory cell; PMEDAP: 9-[2-(phosphonomethoxy) ethyl]-2,6-diaminopurine; PMEG: 9-[2-(phosphonomethoxy) ethyl]-guanine; Hsp90: Heat shock protein 90; HSCT: Hematopoietic stem cell transplant; CSF: Colony stimulating factors; ECIL: European Conference on Infections in Leukemia; GVHD: Graft-versus-host disease; RSV: Respiratory syncytial virus.

## Competing interests

The authors declare that they have no competing interests.

## Authors’ contributions

KA, AB, AM and BS performed the literature research and composed the manuscript. All authors read and approved the final manuscript.
